# Molecular mechanisms of Dicer: endonuclease and enzymatic activity

**DOI:** 10.1042/BCJ20160759

**Published:** 2017-05-04

**Authors:** Min-Sun Song, John J. Rossi

**Affiliations:** 1 Department of Molecular and Cellular Biology, Beckman Research Institute of City of Hope, City of Hope, Duarte, CA 91010, U.S.A.; 2 Irell and Manella Graduate School of Biological Sciences, Beckman Research Institute of City of Hope, City of Hope, 1500 East Duarte Road, Duarte, CA 91010, U.S.A

**Keywords:** endoribonuclease Dicer, microRNA, small interfering RNA, viral small RNA

## Abstract

The enzyme Dicer is best known for its role as a riboendonuclease in the small RNA pathway. In this canonical role, Dicer is a critical regulator of the biogenesis of microRNA and small interfering RNA, as well as a growing number of additional small RNAs derived from various sources. Emerging evidence demonstrates that Dicer's endonuclease role extends beyond the generation of small RNAs; it is also involved in processing additional endogenous and exogenous substrates, and is becoming increasingly implicated in regulating a variety of other cellular processes, outside of its endonuclease function. This review will describe the canonical and newly identified functions of Dicer.

## Introduction

The Dicer enzyme is a member of the ribonuclease (RNase) III family. It is most well known as the endonuclease that functions in the RNA interference (RNAi) pathway to cleave long double-stranded RNA (dsRNA) molecules into short dsRNA molecules, known as small RNAs, including microRNA (miRNA) and small interfering RNA (siRNA). Indeed, Dicer is considered a key factor in the biogenesis of most small regulatory RNAs, and the majority of Dicer studies have focused on this role. However, increasing evidence shows that Dicer also has functions outside of the small RNA pathway. Dicer's endonuclease function is not only involved in small RNA biogenesis, but also in the processing of other endogenous and exogenous substrates. Furthermore, its function is not limited to cleavage, but may regulate other cellular processes. This review will describe the canonical and newly identified functions of Dicer.

## Dicer structure and domains

**Figure 1.**
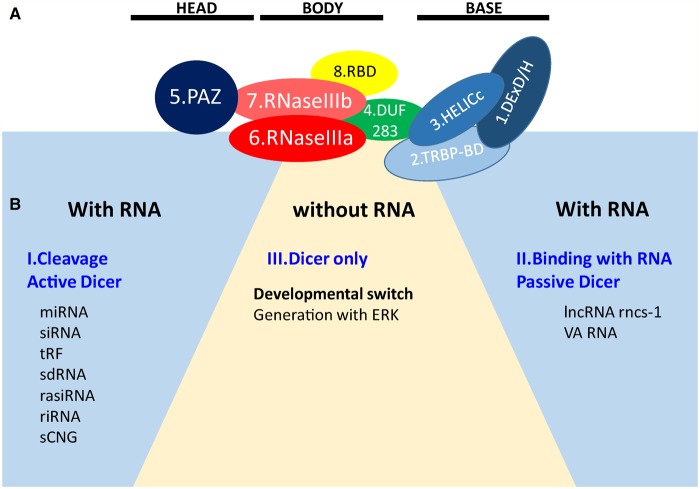
Dicer structure and functions. (**A**) The structure of Dicer. The number one indicates the amino-terminal. The amino-terminal helicase domain forms a clamp-like structure in the base of the L shape and is thought to reorganize and wrap around dsRNA. DExD/H, DExD/H box helicase domain; TRBP-BD, trans-activation response RNA-binding protein-binding domain; HELICc, helicase conserved carboxy-terminal domain; DUF283, domain of unknown function; PAZ, Piwi/Argonaute/Zwille domain; two RNase III domains; RBD, dsRNA-binding domain. (**B**) Multifaceted Dicer function. I. Active Dicer recognizes many types of RNA and can cleave small RNAs. II. Passive Dicer can be stably bound to RNA without endonuclease activity. Dicer does not efficiently process RNA if no free ends are available [33,177] which explains the resistance of the long non-coding RNA rncs-1 against dicing [178]. III. Dicer alone can function as a binding protein. For example, Ras/Erk signaling and Dicer regulate oogenesis, and Dicer was identified as a putative ERK substrate [166].

Although a full-length mammalian Dicer has not been crystallized, many studies have predicted its structure and the positions of its domains and interacting partners [1–5]. The main functional domains of Dicer are ordered from the N- to the C-terminus as follows: helicase domain included with DExD/H, TRBP-BD and HELICc, DUF283, PAZ (Piwi/Argonaut/Zwille) domains, RNase IIIa and IIIb domains, and dsRNA-binding domain (RBD) (Figure 1). Cryo-electron microscopy and crystallography showed that Dicer resembles the shape of the letter L, with a head, a body, and a base [6,7]. At the head is the PAZ domain, which contains binding pockets for the 3′ overhang of a dsRNA substrate. The PAZ domain of Dicer is unique in that it has an extra loop enriched in basic amino acids, which changes the electrostatic potential and molecular surface of the pocket. These differences may affect Dicer RNA binding and handing off the substrate to other protein complexes [8]. The PAZ domain also has a phosphate-binding pocket that recognizes the phosphorylated 5′ end of small RNAs [3]. On the lower half of the Dicer body are the RNase IIIa and IIIb domains, which form the catalytic core of Dicer; each domain is thought to be responsible for the cleavage of one strand of the dsRNA substrate [9]. The DExD/H domain is located in the base of the L and forms a clamp near the RNase III domain active site [1,10]. A recent study showed that the DUF283 domain of Dicer is capable of binding single-stranded nucleic acid [11].

## Evolutionary relationships among Dicer homologs

**Figure 2.**
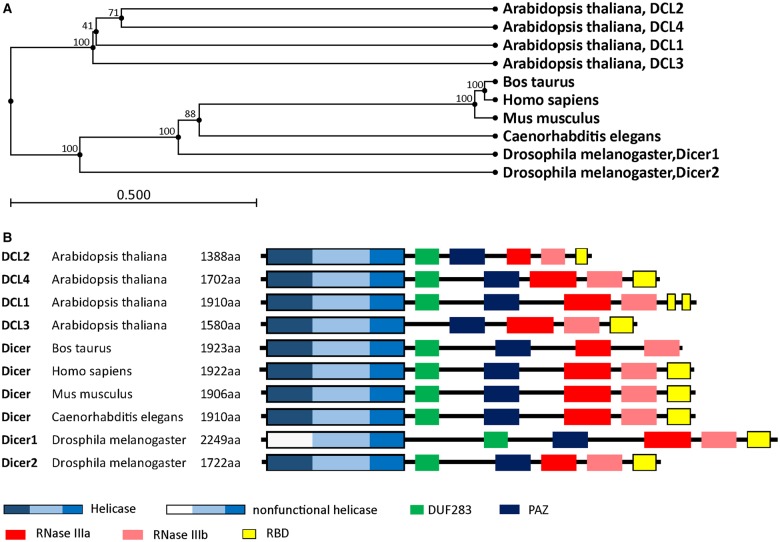
Phylogenetic tree of Dicer and domain analysis. (**A**) Phylogenetic diversity of eukaryotic Dicer proteins. We inferred the Dicer family phylogeny using the maximum likelihood method. The number has been indicated in bootstrap value. (**B**) Complex Dicer protein domain composition. DCL, Dicer-like.

Various numbers of Dicer family proteins can be found in different organisms. Dicer probably arose from an early eukaryotic origin, as it is absent from archaebacteria, but can be found in many eukaryotic organisms, including plants, fungi, and metazoans (Figure 2A) [12,13]. The ancestral RNase III domains are found in eubacteria [14]; but these capabilities were not conserved in eukaryotic Dicer [15]. Dicer has been lost from many parasitic protozoa [16,17] as well as from fungi lacking RNAi, including the model budding yeast *Sccharomyces cerevisiae* [18]. The evolutionary phylogenetic tree of animal Dicers shows that an ancient duplication gave rise to Dicer1 and Dicer2 genes very early in metazoan evolution [19]. Both the miRNA and siRNA pathways rely on a single Dicer protein in vertebrates, and in the Nematoda phylum of invertebrates [9,20]. However, other invertebrates, including the fly *Drosophila melanogaster* and the prawn *Litopenaeus vannamei* of the Arthropoda phylum, and the fluke *Clonorchis sinensis* and the planarian *Schmidtea mediterranea* of the Platyhelminthes phylum retain both the Dicer1 and Dicer2 genes [21]. In *Drosophila*, Dicer1 is essential in the miRNA pathway, while Dicer2 facilitates the siRNA pathway [22]. Consistent with a role in immune defense, Dicer2, the siRNA-dedicated Dicer in Drosophila, is more closely related to the common ancestral Dicer protein than the miRNA pathway-dedicated Dicer1 [23]. This evidence suggests one common Dicer design evolved during metazoan evolution, from a universal factor for the miRNA and siRNA pathways, into a factor specifically adapted for either pathway.

Selective Dicer-mediated cleavage of various RNAs is driven by the evolutionarily conserved helicase domain (Figure 2B) [21,24,25]. Definitive functions for eukaryotic Dicer can be ascribed to different activities within the helicase domain, which is thought to facilitate movement of the protein along long dsRNA molecules [26]. This domain is analogous to the retinoic acid-inducible gene I-like family in the helicase superfamily 2 [27,28]. In *Drosophila*, the helicase domain of Dicer1, which cannot hydrolyze ATP, selectively interacts with the loops of precursor miRNAs (pre-miRNAs) and inhibits cleavage of long dsRNAs [25,29]. In contrast, the helicase domain of Dicer2 requires ATP and processes long dsRNAs to produce siRNAs. In *Caenorhabditis elegans*, Dicer also needs ATP and the helicase domain to process long dsRNAs with blunt or 5′ overhanging termini; mutation of this domain results in weak production of endogenous siRNAs but maintains the ability to dice pre-miRNAs [26,30]. In *Arabidopsis thaliana*, DCL1 mainly facilitates the biogenesis of imperfect stem-loop RNAs into 21 nt miRNAs [31] and also requires ATP [32]. However, although the helicase domains of mammalian Dicer proteins are strongly conserved with *Drosophila* Dicer2 [25], human Dicer does not require ATP [33,34], and cleaves both pre-miRNAs and long dsRNAs [35,36].

## Canonical endonuclease function of Dicer

In the canonical pathway, the biogenesis of most small RNA classes, including miRNAs and many siRNAs, occurs through a stepwise process in the cytoplasm. This process involves association with Dicer and specific members of the large family of Argonaute (AGO) proteins, and assembly into various effector complexes, including the RNA-induced silencing complex (RISC). First, RNAs form a pre-RISC with Dicer [37], aided by the Hsc70/Hsp90 chaperone machinery [38–40]. The RNA is then cleaved by Dicer, in concert with two different dsRNA-binding proteins, the TRBP (HIV trans-activating response RNA-binding protein) and PACT (protein activator of PKR) [41]. After the double-stranded small RNA is captured, one of the two strands is selected as the guide, or active strand, and incorporated into an AGO protein to form the RISC. The duplex RNA is unwound and AGO2 degrades the other strand, known as the passenger strand. In general, the thermodynamic asymmetry of the end of the small RNA duplex is a major factor in determining which strand will be the guide strand; the guide strand is typically the strand with the less stable 5′ end [42,43]. Notably, the guide strand is already determined by the polarity of small RNA duplexes upon loading into the RISC, before unwinding occurs [44–48]. The activated RISC then recognizes a specific target site by complementary intermolecular base pairing throughout the single-stranded guide RNA molecule [49]. The resulting RISC-bound miRNAs or siRNAs either guide the sequence-specific degradation of complementary RNAs or inhibit the translation of partly complementary target messenger RNAs by post-transcriptional gene silencing in the cytoplasm, depending in part on the nature and degree of guide/target complementarity [50,51].

## Additional endonuclease functions of Dicer

In addition, Dicer controls the fate of many other RNA species (Table 1). Dicer-derived small RNAs share several characteristic features. Small RNAs are typically ∼20–30 nucleotides (nt) long; have a characteristic and highly specific structure (i.e. 2-nt 3′ overhangs, and 5′ phosphate and 3′ hydroxyl groups); and contain both passenger and guide strands [24]. This section will describe the role of Dicer in processing a variety of RNA substrates into small RNAs, which together may form a very complex regulatory network.

**Table 1:** Types of endogenous small RNAs generated by Dicer

Origin of small RNA		Small RNA	Mechanism of action
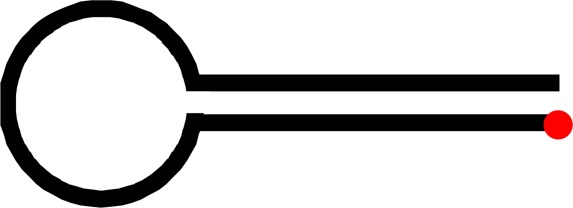	Pre-miRNA	miR320a-3p miR484-3p miR3615-3p miR7706-3p	miRNA biogenesis [176]
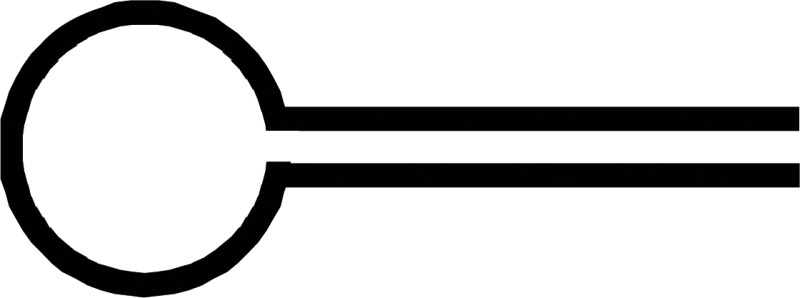	Pre-miRNA	Let-7b,d,g, miR-98, etc.	miRNA biogenesis [176]
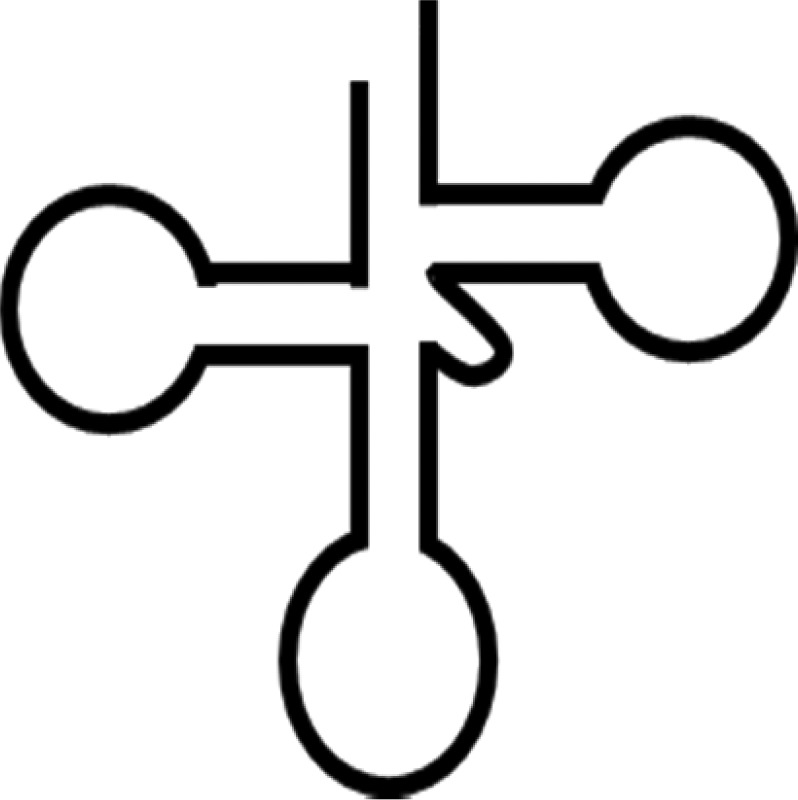	tRNA	5′ tRFs 3′ CCA tRFs	RNAi regulation Translation regulation [53,55–59]
	tRNA	3′ U tRFs	RNAi RNA metabolism [52,54]
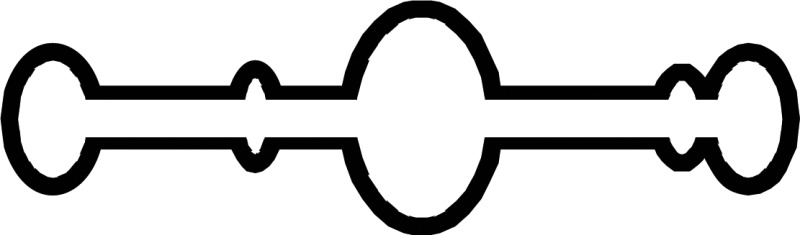	snoRNA	sdRNA	sdRNA RNA metabolism [61–69]
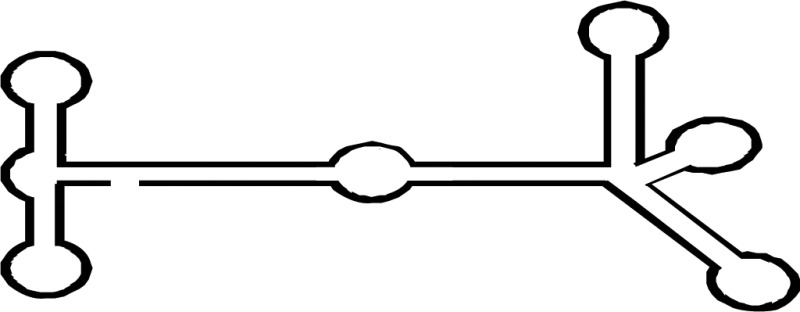	LINE1 SINEs	rasiRNA riRNA	Degradation/repeat-induced small RNA [72–77]
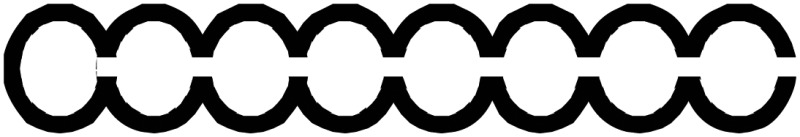	Triplet repeat RNA	sCNG CAG sRNA	Degradation /siRNA [78–81]

Red dot: m^7^ G cap.

### Dicer-derived small RNAs from tRNA

Dicer is important for the production of transfer RNA (tRNA)-related fragments (tRFs), a heterogeneous class of small RNAs. tRFs are generated from tRNAs, and high-throughput sequencing and analysis show that they map to known tRNA genes [52–54]. The most abundant forms of tRFs can be classified into several groups. Three types of tRFs are derived from mature tRNA or pre-tRNA: 3′ U tRFs (tRF-1), 5′ tRFs (tRF-5), and 3′ CCA tRFs (tRF-3). 3′ U tRFs are generated by tRNA 3′-endonuclease ELAC2 (RNaseZ) [52]. 5′ tRFs are produced from the 5′ end of the tRNA generated at any point of tRNA processing, provided the 5′ leader sequence is removed by RNaseP, and are formed by a cleavage in the D loop. 3′ CCA tRFs are derived from the 3′ ends of mature tRNA by cleavage at the T loop and carry the trinucleotide CCA at the acceptor stem. Both 5′ tRFs and 3′ CCA tRFs have 5′ phosphate and 3′ hydroxyl ends, similar to miRNAs, and can be cleaved by Dicer protein complexes [53,55–58].

3′ CCA tRFs are processed in a Dicer-dependent manner [53]. Both *in vitro* and *in vivo* studies show that tRFs derived from human tRNA(Gln) are dependent on Dicer [53,55]. The tRNA(Gly)-derived tRF has been suggested to have miRNA-like functions. Dicer is implicated in the generation of CU1276, a 22-nt tRNA(Gly)-derived small RNA expressed in mature B cells, which is physically associated with AGO and represses an endogenous target mRNA. It inhibits the mRNA of the single-stranded DNA-binding protein RPA1, which suppresses cell proliferation and modulates the DNA damage response [58]. The miRNA biogenesis pathway utilizes tRNA processing enzymes and is found in specific herpesviruses. Murine γ-herpesvirus 68 miRNA encodes a viral tRNA-like sequence transcribed by RNA polymerase III to generate tRNA-like primary miRNA precursors (pri-miRNAs). Pri-miRNAs carry a 5′ tRNA moiety and are cleaved by a cellular tRNaseZ. Dicer processes the 3′ tRNA moiety to generate mature viral small RNAs [59].

The lupus autoantigen La is an RNA chaperone associated with the miRNA pathway. La functions as a gatekeeper that ensures correct tRNA maturation and protects the miRNA pathway from tRF. In the absence of La (La knockdown cells), various pre-tRNAs bind to Dicer, and the tRFs derived from the 3′ ends of tRNAs are bound to AGO [60].

### Dicer-derived small RNAs from non-coding RNA

Dicer plays a role in processing several types of non-coding RNAs, which has implications for various regulatory mechanisms.

#### Dicer and snoRNA

Processing of non-coding small nucleolar RNA (snoRNA) to shorter, more stable snoRNA-derived RNAs (sdRNAs) is widespread, with sdRNAs identified in vertebrates, including humans [61–68], plants [63], fission yeast [63], and protozoa [69]. A high-throughput sequencing study of human small RNAs demonstrated that specific processing led to accumulation of small RNAs emerging from well-characterized non-coding RNAs, including snoRNAs [61]. sdRNAs are associated with AGO proteins and show miRNA capabilities, which may have implications for control of mRNA processing and translation [62,69]. Association with Dicer varies by type; many sdRNAs derived from box H/ACA snoRNAs are processed by Dicer, while processing of box C/D snoRNAs can be Dicer-independent [57,62]. In contrast, knockdown of Dicer and AGO in *Giardia lamblia* (protozoa) led to identification of small fragments with miRNA characteristics and showed that Dicer is required for miR2 production from box C/D snoRNA [69]. The identification of small fragments derives from the scaRNA of ACA45 (SCARNA15) by deep sequencing of human small RNAs associated with AGO proteins. The scaRNAs are a specific class of snoRNA that localize to Cajal bodies and guide the modification of RNA polymerase II transcribed spliceosomal small nuclear RNAs (snRNAs) U1, U2, U4, and U5 [70]. ACA45 is a 108-nt double-hairpin RNA that can be processed by Dicer to generate 20- to 22-nt ACA45 small RNA products [62].

A lot of data support the hypothesis that Dicer has direct roles in post-transcriptional gene-silencing activity of many miRNA-like sdRNAs, but the mechanism underlying their biogenesis remains unknown. It has been shown that processing of box C/D RNAs into sdRNAs can be Dicer-independent, while production of box H/ACA-derived sdRNAs requires Dicer [57,62]. Dicer is able to process dsRNA from snoRNAs [54,62,63,71]. Thus, snoRNAs can serve as a source of short regulatory RNA species generated by Dicer, which may be involved in the control of processing and translation of various mRNAs.

#### Dicer and long interspersed nuclear element-1

Another example of non-coding RNA processing by Dicer involves LINE-1 (long interspersed nuclear element-1), a retrotransposon. The LINE-1 promoter has a bidirectional orientation, containing sense and antisense transcripts that form double-stranded long hairpin RNAs, which are then processed by Dicer into repeat-associated siRNAs (rasiRNAs). rasiRNAs are loaded into AGO proteins to silence LINE-1 by RNA-directed DNA methylation. Inhibition of biogenesis of rasiRNAs has been suggested to increase the processing of LINE-1 transcription via Dicer inhibition [72]. Other experiments using engineered LINE-1 reporters have shown that mutation of Dicer increases LINE-1 transcripts. The rasiRNAs generated by Dicer negatively regulate LINE-1 transcription at the post-transcriptional level [73,74].

#### Dicer and short interspersed element

Geographic atrophy (GA), an age-related macular degeneration disease of the retinal pigment epithelium, is associated with reduced expression of Dicer. Inhibition of Dicer leads to accumulation of Alu repeat RNAs of ∼300-nt derived from short interspersed elements (SINEs). This accumulation is cytotoxic and triggers interferon-mediated, caspase 8-dependent apoptosis. Injection of Alu repeat RNA into mouse eyes generates GA [75]. This condition is rescued by Dicer cleavage of Alu repeat RNAs, suggesting that Dicer-dependent degradation of the RNAs is critical for detoxification [75]. Dicer processing of Alu repeat RNA prevents activation of the host inflammasome [76]. The DR2 Alu repeat RNAs are processed into 28–65-nt repeat-induced small RNAs (riRNAs) under DCIER-dependent conditions. These riRNAs are stabilized by binding with AGO3 and recruit mRNA-decapping complexes, which block translation and degrade key stem cell transcripts like Nanog and Tdgf1 mRNAs [77].

### Dicer-derived small RNAs from triplet repeat RNA

Dicer has direct and indirect roles in curtailing expression of toxic triplet repeat RNAs. Triplet repeat expansion disorders include myotonic dystrophy type 1 (DM-1), fragile X-associated tremor ataxia syndrome, Huntington's disease (HD), and spinocerebellar ataxia (SCA), which frequently encode RNAs with long internal triplet repeat structures [78]. *In vitro* studies showed that single-stranded CGG-RNA is cleaved by Dicer to generate short CGG-RNAs [79]. Dicer recognizes and processes the hairpin structure of expanded CNG repeats, producing small CNG-repeated RNAs (sCNG) [78,80]. The Dicer-dependence of sCNG biogenesis has been demonstrated in fibroblasts of patients with DM-1 (sCUG), HD (sCAG), and SCA (sCAG) [78]. The relevance of sCNG has been reported in HD, which is caused by an abnormal CAG expansion within the first exon of the Huntingtin (HTT) gene. The sCAGs from HTT RNA stimulate neurotoxic activity [81]. Importantly, the toxic activity of sCAG is dependent on Dicer.

### Dicer-derived small RNAs from exogenous RNA

In addition to its role in processing endogenous RNA, Dicer is also important in processing exogenous RNA, including viral-associated (VA) RNA (Table 2). Viral infection can produce virus-derived small RNAs. For example, VA RNA I and II are 160-nt long non-coding RNAs from the adenovirus genome, which are processed similarly to miRNAs, resulting in the production of 22-nt VA-RNA-derived miRNAs (mivaRNAs). These mivaRNAs are processed by Dicer [82–84].

**Table 2:** Types of exogenous small RNAs generated by Dicer

Origin of small RNA		Small RNA	Mechanism of action
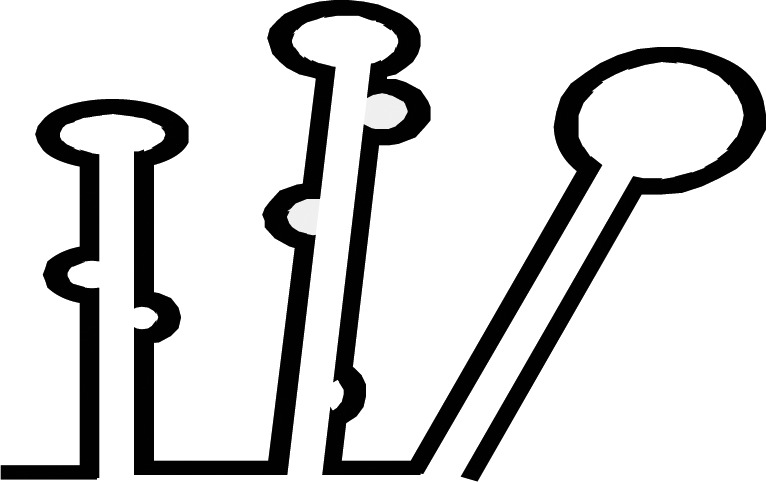	Adenovirus VA RNA	mivaRNA	Antiviral response [82–84]
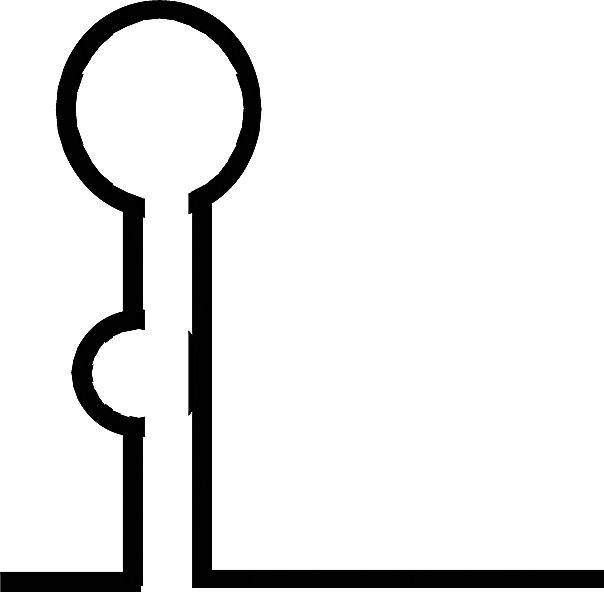	HIV TAR RNA	miR-N367	Antiviral response [88–91]

RNA genome viruses do not express small RNA in infected cells; this phenomenon is believed to be a mechanism of host immune evasion [85,86]. However, the bovine leukemia virus, a retrovirus with an RNA genome, encodes a conserved cluster of small RNAs that are produced as shorter RNA polymerase III-transcribed hairpins that are directly cleaved by Dicer [87].

Recent studies show that RNA viruses also process miRNA-like RNAs [88,89]. A small RNA within the Nef (negative regulatory factor) region of the human immunodeficiency virus type 1 (HIV-1) genome is proposed to play a role in inhibition of viral transcription [90]. A 19-nt small RNA is derived from hairpin structures, including TAR (trans-activation response element) RNA [91]. Dicer processes these structures into functional small RNAs that target the HIV-1 genome. The Nef-derived miRNA (miR-N367) reduces Nef expression and suppresses replication by reducing HIV-promoter activity.

## Additional functions of Dicer

In addition to the critical role of Dicer in small RNA biogenesis, it also has important roles in genome regulation and surveillance, unconnected to small RNA production. For example, although its RNAi role occurs in the cytoplasm, Dicer can also localize to the nucleus [92,93] and interact with nuclear pore components [94]. Dicer is present in autophagy and is required for autophagosome formation [95–97]. Dicer is found in the exosomes that are tiny vesicles with RNAs and proteins that are secreted from cells [98,99]. Dicer can also bind to ‘passive' RNA-binding sites without cleavage or small RNA generation [100]. Such interactions act as a buffering system to stabilize passive-site RNAs and control the catalytic activity of the enzyme by sequestering it from other targets. Dicer can also process viral sources of dsRNAs to produce viral siRNAs (viral small interfering RNAs) involved in various antiviral silencing responses [101].

### Dicer in the nucleus

Several lines of evidence suggest that Dicer plays important roles in the nucleus. The C-terminal dsRNA-binding domain of Dicer contains a nuclear localization signal, which allows it to shuttle between the nucleus and the cytoplasm [93]. Human Dicer is associated with a nuclear pore complex component, nucleoporin 153, which assists Dicer in transport and localization to the nucleus [94]. Recent studies have shown that mammalian Dicer can also function in the nucleus [102]. Dicer inhibition leads to altered nucleolar structure; a significant increase in nucleolar size was observed in cells depleted of Dicer [103]. Some studies show that Dicer is required for cleavage and processing of pre-ribosomal RNA (rRNA) [104,105], which occurs mainly in the nucleolus [106], suggesting a connection between nuclear Dicer, rRNA synthesis, and ribosome biogenesis. However, another study shows that Dicer is associated with rDNA, but did not process pre-rRNA [7]. This suggests Dicer may affect pre-rRNA processing in different ways, such as transcriptional regulation.

Nuclear siRNAs corresponding to heterochromatic loci are associated with silencing; Dicer and AGO1 mutations destroy the production of these endo-siRNAs and the assembly of heterochromatin in the fission yeast *Schizosaccharomyces pombe* [107,108]. These siRNAs produce sequence-dependent silencing by recruiting histone-modifying enzymes that initiate heterochromatin formation and correlate with AGO1 and other silencing components [107,109]. Such siRNA-mediated transcriptional silencing has been reported in *Arabidopsis* [110,111], *Drosophila* [112], and mammalian cells [113]. In mammalian cells, nuclear siRNA-mediated transcriptional gene silencing was induced by histone deacetylases and DNA methyltransferases [113]. Another study showed heterochromatin defects at centromeres in Dicer-inhibited cells [114], suggesting that the Dicer-related RNAi machinery is implicated in the formation of the heterochromatin structure. Recent studies show that mammalian Dicer can be located in the nucleus [102]. The nuclear localization and function of mammalian Dicer remains one of the least understood aspects of Dicer biology.

### Dicer in exosomes

Exosomes are small extracellular vesicles with an average size of 30–100 nm, which were simply considered cell debris for many years. Today, exosomes are known to be rich in lipids, proteins, and RNAs, including protein-coding transcripts (i.e. mRNAs) as well as many types of non-coding RNAs (e.g. miRNAs, long non-coding RNAs, circular RNAs, snoRNAs, snRNAs, tRNAs, rRNAs, and piwi-interacting RNAs) [115]. Exosomes allow these RNAs to be transferred from parent cells to recipient cells, where they can regulate or serve as templates for protein production [116,117].

In addition to recognition of a novel form of cell–cell communication, the discovery of miRNAs in exosomes has suggested new applications for exosomes, such as potentially easy-access biomarkers, or, given that exosomes are non-immunogenic, possibly as novel therapeutics [118–124]. miRNAs transported via exosomes could function as gene expression regulators in recipient cells, and growing evidence from exosomes in cancer and other processes has expanded the known subsets of exosome functions [121,125–128]. The discovery of miRNAs in exosomes also suggests a mechanism through which dysregulation of endogenous miRNAs may affect disease pathogenesis.

Dicer, AGO2, and TRBP proteins are detected in exosomes derived from breast cancer cells, but not from non-tumorigenic breast cells [98]. Exosomes derived from cancer cells, but not normal cells, were enriched in mature miRNAs, suggesting that pre-miRNAs can convert into mature miRNA in exosomes from cancer but not from non-tumorigenic breast cells. Tumor-derived exosomes isolated from cells or fluids of patients with breast cancer can be transferred to non-cancer cells in a Dicer-dependent manner. Mice form tumors when injected with cancer-derived exosomes, except in the presence of Dicer inhibition. Thus, Dicer is important for the transformation of normal cells to cancer cells, following exposure to exosomes. Exosomes carrying Dicer-derived miRNAs can affect cell–cell communication. Dicer-sufficient macrophage-derived exosomes carry miRNAs into Dicer-deficient endothelial cells to measurably reduce targeted sequences [99]. Thus, exosomes released by cancer cells may bioengineer miRNAs with the assistance of Dicer, resulting in tumor boosters.

Exosomes derived from HIV-1-infected cells contain TAR RNA and Dicer, as well as components of the host miRNA machinery [129]. The inclusion of TAR RNA and these components requires CRM1 transport receptor-mediated export from the nucleus; after packaging, the viral exosomes are released into the extracellular space. In recipient cells, exosomal TAR RNA down-regulates apoptosis by reducing expression of pro-apoptotic protein Bim and transcriptional regulator cyclin-dependent kinase 9. Thus, viral exosomes may provide a mechanism for intercellular viral spread in HIV-infected hosts.

### Dicer DNase function

Dicer also plays a role in the recognition and processing of DNA in apoptosis [130]. The caspase CED-3 cleaves the two RNase III domains of Dicer in *C. elegans*, leaving a truncated though catalytically active protein consisting only of the C-terminal RNase III and dsRNA-binding domains. This truncated Dicer inactivates its RNase function, gains DNase function, and initiates breaks on chromosomal DNA. The DNA fragments are metabolized to complete apoptotic DNA degradation.

## Two faces of Dicer viral interactions

### Dicer antiviral function

Antiviral RNAi is a powerful mechanism to protect against viral infections. Specific features of viral RNA are sensed as foreign, and Dicer cleaves the double-stranded viral RNA into viral siRNA [10,131–134]. As described above, VA RNAs are processed by Dicer into small RNAs (mivaRNAs). These are incorporated into the RISC and may play an important role in suppressing RNAi or miRNA regulation during viral infection. VA RNA I is quickly generated after infection of host cells and amasses high copy numbers to support adenovirus survival [135–138]. VA RNA I has RNA-silencing properties, in addition to its well-established role in blocking dsRNA-dependent protein kinase PKR activation [139–141]. Activated PKR is designed to stop translation in virus-infected cells by phosphorylating a translation initiation factor [84]. VA RNA is a highly structured RNA that is bound by the nuclear export protein Exportin 5. It is rapidly transcribed after infection and produces very high copy numbers. VA RNA I saturates the Exportin 5 pathway, thereby inhibiting the nuclear export of pre-miRNAs. Additionally, due to its pre-miRNA-like secondary structure, VA RNA I suppresses Dicer mRNA movement to the cytoplasm by saturating the Exportin 5-dependent export pathway [139,142]. On the other hand, VA RNA I plays an important role in blocking antiviral function, thereby inhibiting Dicer mRNA. This suggests that Dicer has roles in antiviral function.

Although VA RNA is a poor substrate for Dicer cleavage, a few VA RNAs are cleaved to ∼22-nt mivaRNAs. mivaRNA association with the RISC is crucial for adenovirus replication [143]. A recent study showed that mivaRNAs down-regulate adenovirus replication, and knockdown of Dicer significantly promotes adenovirus replication by inhibiting production of mivaRNAs [82]. This suggests that Dicer performs its antiviral function against adenovirus via cleavage of VA RNAs.

Vaccinia virus (VACV) infection shows that inhibition of Dicer protein is associated with reduction in pre-miRNA processing [144]. Flaviviruses produce a small pathogenic RNA called subgenomic flavivirus RNA (sfRNA), which suppresses RNA silencing in insect and mammalian cells. The sfRNAs are associated with Dicer in infected cells and compete with endogenous small RNAs, resulting in decreased Dicer activity [145–148]. The human herpesvirus Epstein–Barr virus (EBV)-derived miRNA miR-BART6-5p targets human Dicer mRNA [149]. This suggests that viral miRNA influences Dicer transcription via a more subtle mechanism. This phenomenon shows the competition between Dicer of host cells and viral miRNAs of exogenous RNA.

EBV miRNA can be edited by adenosine deaminase acting on RNA to convert adenosine into inosine. A-to-I-edited miR-BART6-3p decreases the efficiency with which the miRNA encoded on the opposite strand, miR-BART6-5p, is loaded into the RISC in human cells. miR-BART6-5p targets human Dicer though four binding sites in its 3′UTR. Therefore, editing of miR-BART6-3p has a positive effect on Dicer expression. In turn, Dicer levels affect the expression levels of multiple genes that regulate the infectious and lytic states of EBV. Thus it is postulated that editing of miR-BART6-3p could be an indirect way to modulate miRNA biogenesis and thereby the viral life cycle [149].

Viruses encode proteins that suppress RNAi in mammalian cells, e.g. E3L (VACV), B2 (Nodamura virus), NSs (La Crosse virus), Tat (HIV), and VP30, VP35, and VP40 (Ebola virus) [142,150–154]. In particular, B2 of the Nodamura virus binds exogenous short hairpin RNAs and endogenous pre-let-7d and inhibits Dicer small RNA biogenesis [152]. The helicase domain of Dicer interacts with the lysine 51 residue of HIV Tat. The interaction between Tat and Dicer is dependent on the presence of small RNA, as RNase treatment inhibits this interaction. Tat interferes with Dicer processing of miRNA biogenesis [91]. VP30 and VP35 can function through direct interaction with Dicer or Dicer-associated factors TRBP and PACT, and inhibit the production of functional small RNAs [153,154]. These results demonstrate that viral RNA-binding proteins have the potential to interfere with miRNA biogenesis through RNA–Dicer interactions.

### Dicer proviral function

Virus-derived small RNAs that direct specific antiviral defenses through biogenesis of a small RNA may be dependent on the cleavage activity of Dicer. However, it is still under debate whether infection by virus induces or suppresses antiviral small RNA.

*A. thaliana* encodes multiple Dicer proteins (DCL 1–4) involved in distinct endogenous RNA-silencing pathways and antiviral defenses, which show functional redundancy and specificity [155,156]. Inactivation of DCL4 but not DCL2 or DCL3 induces a high level of viral replication, indicating that DCL4 is essential for intracellular antiviral silencing. While DCL2 can produce abundant 22-nt viral siRNAs when DCL4 is absent, these siRNAs are less efficient in mediating an antiviral defense [157,158]. As described above, Dicer has two types in *Drosophila*. Dicer1 produces mature miRNAs in an ATP-dependent manner. Dicer2 cleaves cell-derived dsRNA precursors to produce endogenous siRNAs [159] and exogenous siRNAs [160,161], and mediates host defenses against viruses by activating Toll immune signaling [160]. The specific type of Dicer that processes virus-derived dsRNA into small RNAs in *A. thaliana* and *D. melanogaster* suggests an antiviral strategy, but other Dicer types do not produce antiviral small RNAs from viruses. In contrast, humans have one type of Dicer that processes both miRNAs and siRNAs. These various roles of Dicer led us to hypothesize that Dicer might regulate both antiviral and proviral mechanisms.

Infection of human cells with a wide range of viruses, including yellow fever virus, Sindbis virus, Venezuelan equine encephalitis virus, measles virus, influenza A virus, reovirus, vesicular stomatitis virus, HIV-1, or herpes simplex virus 1, failed to reveal any enhancement in viral replication in Dicer-inhibited human cells (human cells with the absence of Dicer) [162]. miRNAs derived from the Dengue virus genome include six different miRNA-like molecules (viral small RNA: vsRNA 1–6) [163]. Inhibition of vsRNA-5 leads to significant increases in viral replication. However, inhibition of the other vsRNAs has no direct effect on viral replication. Importantly, viral replication proceeded more slowly in human Dicer-deficient cells. KUN-miR-1 is derived from the terminal 3′ stem loop of West Nile virus (WNV) [164], and its production reduces inhibition of Dicer, with a significant reduction in WNV replication.

## Dicer as a developmental switch

The Dicer protein can command specific interactions with other proteins. For example, Ras/ERK signaling and Dicer regulate *C. elegans* oogenesis; Dicer is a putative ERK substrate [165]. Phosphorylation of Dicer by ERK localizes it to the nucleus; it remains present during most of oogenesis but is rapidly lost from the terminal oocyte shortly before fertilization [166]. An ERK-mediated switch in Dicer activity could regulate genes during oogenesis and in the oocyte–embryo transition.

## Dicer and cancer

In addition to other diseases described throughout this review, many groups have reported a correlation between Dicer expression and cancer. For example, Dicer expression shows significant changes in different stages of lung adenocarcinoma [167]. Reduced expression of Dicer is associated with poor prognosis in some types of lung, breast, skin, endometrial, and ovarian cancer [168–172]. In contrast, Dicer is overexpressed in metastatic lesions of prostate cancer [173], and is increased in Burkitt lymphoma [174]. There is no clear correlation between Dicer expression, cancer type, and disease progression; whether these inconsistencies can be attributed to technical artifacts, to tissue specificity, or to some other biological process remains unknown. Thus, whether Dicer acts as a tumor suppressor or an oncogene remains controversial [175]. However, given Dicer's dual roles in other biological processes, one can speculate that Dicer may function as both a tumor suppressor and an oncogene.

## Conclusions

Accumulating evidence continues to demonstrate the critical role of the Dicer enzyme in not only the biogenesis of miRNA and siRNA in the canonical RNAi pathway, but also in controlling the fate of many other small RNA species. Furthermore, the list of non-endonuclease roles of Dicer continues to grow. Future directions in Dicer research should include: expanding our understanding of Dicer in the biogenesis and regulation of a growing network of endogenous small RNAs, which have interrelated implications for the regulation of complex physiological processes, including cancer; delineating the regulation of Dicer itself, including the causes and consequences of Dicer subcellular localization; and determining the role of Dicer in response to exogenous RNAs, and how this connects to the immune system.

## Abbreviations

AGO: Argonaute
CRM: chromosome region maintenance
DCL: Dicer-like
DExD/H: DEAD-like and helicase domain
DM-1: myotonic dystrophy type 1
dsRNA: double-stranded RNA
EBV: Epstein–Barr virus
ERK: extracellular signal-regulated kinase
GA: geographic atrophy
HD: Huntington's disease
HIV-1: human immunodeficiency virus type 1
HSV: herpes simplex virus
HTT: Huntingtin
LINE-1: long interspersed nuclear element-1
miRNA: microRNA
mivaRNAs: VA-RNA-derived miRNA
Nef: negative regulatory factor
PACT: protein activator of PKR
PAZ: Piwi/Argonaut/Zwille
PKR: RNA-dependent protein kinase
pre-miRNAs: precursor miRNAs
pri-miRNAs: primary miRNA precursors
rasiRNA: repeat-associated siRNA
RBD: dsRNA-binding domain
riRNA: repeat-induced small RNA
RISC: RNA-induced silencing complex
RNAi: RNA interference
RNase: ribonuclease
rRNA: ribosomal RNA
SCA: spinocerebellar ataxia
scaRNA: Cajal body- specific RNA
sCNG: small CNG
sdRNA: snoRNA-derived RNAs
sfRNA: subgenomic flavivirus
sfRNA: subgenomic flavivirus RNA
SINE: short interspersed elements
snoRNA: small nucleolar RNA
snRNA: small nuclear RNAs
TAR: trans-activation response element
TRBP: HIV trans-activating response RNA-binding protein
tRF: transfer RNA-related fragments
tRNA: transfer RNA
VA RNA: viral-associated RNA
VACV: vaccinia virus
viral siRNA: viral small interfering RNA
vsRNA: viral small RNA
WNV: West Nile virus.
